# Retinal Changes Induced by Epiretinal Tangential Forces

**DOI:** 10.1155/2015/372564

**Published:** 2015-09-03

**Authors:** Mario R. Romano, Chiara Comune, Mariantonia Ferrara, Gilda Cennamo, Stefano De Cillà, Lisa Toto, Giovanni Cennamo

**Affiliations:** ^1^Dipartimento di Neuroscienze, Scienze Riproduttive ed Odontostomatologiche, University Federico II, Napoli, Italy; ^2^Azienda Ospedaliero-Universitaria “Maggiore della Carità” di Novara, 28100 Novara, Italy; ^3^Ophthalmology Clinic, University G. d'Annunzio of Chieti-Pescara, 66100 Chieti, Italy

## Abstract

Two kinds of forces are active in vitreoretinal traction diseases: tangential and anterior-posterior forces. However, tangential forces are less characterized and classified in literature compared to the anterior-posterior ones. Tangential epiretinal forces are mainly due to anomalous posterior vitreous detachment (PVD), vitreoschisis, vitreopapillary adhesion (VPA), and epiretinal membranes (ERMs). Anomalous PVD plays a key role in the formation of the tangential vectorial forces on the retinal surface as consequence of gel liquefaction (synchysis) without sufficient and fast vitreous dehiscence at the vitreoretinal interface. The anomalous and persistent adherence of the posterior hyaloid to the retina can lead to vitreomacular/vitreopapillary adhesion or to a formation of avascular fibrocellular tissue (ERM) resulting from the proliferation and transdifferentiation of hyalocytes resident in the cortical vitreous remnants after vitreoschisis. The right interpretation of the forces involved in the epiretinal tangential tractions helps in a better definition of diagnosis, progression, prognosis, and surgical outcomes of vitreomacular interfaces.

## 1. Introduction

Two kinds of forces are involved in vitreoretinal traction diseases: tangential and anterior-posterior forces. The formation of these forces is due to the anomalous posterior vitreous detachment. When the posterior hyaloid partially detaches from the posterior pole and macula, keeping a strong attachment at a focal point, the anterior-posterior vector appears. The tangential forces result from vectors that are tangent to the retinal surface. These forces are less characterized and classified in literature compared to anterior-posterior ones, although they are responsible for retinal damages of the inner and outer layers [1]. We will characterize the tangential vectors involved in the retinal changes: (i) anomalous posterior vitreous detachment and vitreoschisis; (ii) epiretinal membranes (ERMs); (iii) vitreopapillary adhesion (VPA).

## 2. Anomalous Posterior Vitreous Detachment and Vitreoschisis

**Figure 1:**
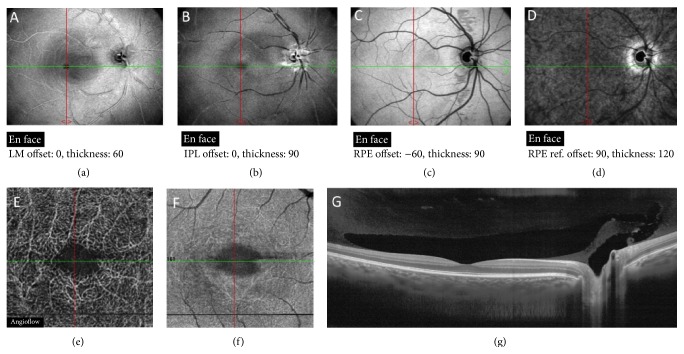
OCT images of normal eye. (a)–(d) En face OCT images at different segmentation thicknesses. (e)-(f) Angio-OCT images of, respectively, superficial and deep retinal plexus. (g) Premacular bursa appears on OCT as boat-shaped lacunae in the macular region.

The vitreous is an extended extracellular matrix that is composed mainly of water (98%); in youth, it is nonetheless a solid gel due to the intricate association of hyaluronan, collagen, and additional molecular components [2]. In youth, this fine structure is strongly adherent to the retina in a fascial (as opposed to focal) manner [3], although the exact processes and cells responsible for the synthesis and adhesion of such macromolecules remain unidentified [4]. The main function of the vitreous, however, is the maintenance of transparency within the eye (Figure 1). This feature minimizes light scattering, allowing the unhindered transmission of photons to the retina for photoreception [5, 6]. During aging, changes in vitreous macromolecular interactions result in the formation of a liquid vitreous that consists primarily of hyaluronan and water as well as collagen fibrils that aggregate into bundles of parallel fibers [7]. Moreover, during aging, changes at the interface weaken vitreoretinal adhesion and promote vitreoretinal dehiscence in most individuals. These two processes must occur in concert and simultaneously to result in an innocuous posterior vitreous detachment (PVD) [8]. Posterior vitreous detachment is characterized by synchysis (gel liquefaction) and syneresis (vitreoretinal dehiscence with collapse) of the posterior vitreous away from the retina [4]. The effect of vitreous syneresis varies from negligible to significant, depending on the condition of the vitreoretinal interface [9]. In diseases such as age-related macular degeneration [10] and diabetic retinopathy [11], complete PVD is less frequent, but, if present, it protects against more advanced stages of disease [12].

### 2.1. The “Anomalous PVD”

In a minority of cases, a clean separation of the posterior vitreous cortex (PVC) from the retina does not occur due to the presence of liquefaction without sufficient dehiscence at the vitreoretinal interface. This is known as “anomalous PVD” [13]. Five stages of PVD have been described: stage 0, absence of PVD; stage 1, focal perifoveal PVD, with persistent attachment to the fovea, optic nerve head, and midperipheral retina; stage 2, macular PVD with persistent attachment to the optic disc but without vitreofoveal adhesion; stage 3, near-complete PVD with VPA only; and stage 4, complete PVD [14, 15]. The sequelae of anomalous PVD vary depending on the position of the strongest retinal adherence of the PVC and greatest liquefaction of the gel. In the peripheral fundus, advanced gel liquefaction with firm vitreoretinal adhesion causes retinal detachments and tears [8]. At the optic disc, anomalous PVD can cause different vitreopapillopathies, and it can also play a role in promoting vitreous hemorrhage and neovascularization in ischemic retinopathies [8]. At the macula, anomalous PVD can induce various pathologies, and it is relevant if the PVC that remains adherent to the retina is of partial thickness or full thickness. Peripheral vitreoretinal separation with full-thickness vitreous cortex adherence to the macula can induce vitreomacular traction (VMT). In presence of symptomatic intraretinal changes, this condition is known as vitreomacular traction syndrome (VMTS) [8]. In VMTS, a broad, full-thickness posterior vitreous adhesion occurs to the margin of the fovea (macular adhesion size of ~ ≥1500 *μ*m) [16]. Meanwhile, a more focal adhesion induces vitreofoveal traction syndrome (foveal adhesion size < 500 *μ*m) [16]. However, the smaller the diameter of the vitreofoveal adhesion is, the greater the tractional force that is exerted is, causing more serious foveal deformation [16]. In these instances, peripheral vitreoretinal separation in the presence of persistent adhesion at the macula is evident. The tractional forces are mainly anteroposterior, causing a central cyst in the vitreofoveal traction and retinal thickening with edema in the VMTS [8].

### 2.2. Vitreoschisis

Vitreoschisis is a consequence of a splitting in the PVC [8]. The PVC has a multilamellar structure, composed of densely packed collagen fibrils that run parallel to the retinal surface. It is comprised mostly of type II collagen, but a hybrid of types V/XI and type IX is also present [13, 17]. The adhesion molecules such as fibronectin, laminin, and heparan sulfate keep these collagen fibers attached to the retina surface and interact with opticin in the vitreous gel [18, 19]. The packed collagen fibrils of the PVC are superficially inserted into the internal limiting membrane (ILM) of the retina [17]. The PVC is 100–300 *μ*m in thickness; it is thinnest at the fovea, where collagen fibrils are more densely packed [17]. Hyalocytes, resident mononuclear phagocytes, are located in a single layer in the PVC approximately 50 *μ*m from the ILM of the retina [20–22].

Vitreoschisis results from anomalous PVD [13] in presence of a firm vitreomacular adhesion (VMA) that causes a splitting of the PVC during syneresis, in which the outermost vitreous layer remains attached to the macula, while the remaining vitreous collapses forward [8].

Vitreoschisis induces various vitreomacular pathologies [8]. Kakehashi and colleagues detected vitreoschisis in patients with retinovascular diseases by applying clinical biomicroscopy [23]. Studies using ultrasonography [24] and histopathology [25] have also resulted in the diagnosis of vitreoschisis in proliferative diabetic retinopathy. Recently, combined optical coherence tomography/scanning laser ophthalmoscopy (OCT/SLO) led to the clinical diagnosis of vitreoschisis. In vitreoschisis, two membranous layers appear to join into one, forming the shape of the letter “Y” or “lambda” (*λ*); in other cases, this shape is not observed, but there is visible evidence of a membrane attached to the retina and a separate and distinct second membrane on the posterior side of the detached vitreous [26]. Indeed, studies [27] conducted at the VMR Institute using combined OCT/SLO imaging have identified vitreoschisis in 53% of the patients with macular holes (MHs) and in 43% of the patients with macular pucker (MP). These studies have suggested that it is important whether the split occurs anteriorly or posteriorly to the level of the hyalocytes [20–22]. If the split occurs posteriorly to the layer of hyalocytes, a thin hypocellular membrane remains attached to the macula. If this membrane also maintains its attachment to the optic disc, it may cause an outward (centrifugal) tangential contraction, inducing a MH. If the split occurs anteriorly to the layer of hyalocytes, the remaining layer of vitreous attached to the macula will include the hyalocytes and will be relatively thick, hypercellular, and contractile. As mononuclear phagocytes of the reticuloendothelial cell system, “sentinel” hyalocytes can stimulate the migration of glial cells from the circulation to the retina and monocytes [26]. Contraction of this tissue induces inward (centripetal) tangential traction upon the underlying retina, causing a MP. Recent studies [28] have shown that cytokines can influence hyalocyte metabolism and may also induce further cell proliferation; furthermore, other studies [29] have demonstrated that hyalocytes can cause collagen gel contraction in response to platelet-derived growth factor (PDGF) and other cytokines. Hence, hyalocytes are likely to be important in stimulating cell proliferation and in inducing tangential vitreoretinal contraction.

The hypothesis that the PVC can split into layers is fundamental to the theory of anomalous PVD with vitreoschisis [8]. The collagen fibrils within the PVC have a multilamellar organization, which has been confirmed in studies performed in monkeys [26, 27, 30], and these lamellae constitute a series of potential cleavage planes. The splitting of the PVC can also occur during vitreous surgery. Yamashita et al. were the first investigators to present in vivo intraoperative evidence of the occurrence of vitreoschisis [31]. They noted that 80% of eyes with MP develop vitreoschisis during vitrectomy surgery. Among those eyes in which vitreoschisis occurred intraoperatively, 58% had a visible “hole” in the PVC, while the remaining 42% had no visible hole. This finding suggests that vitreoschisis can occur at various levels within the PVC, consistent with the underlying multilamellar anatomy of the PVC [31]. These results could influence the performance of vitreoretinal surgery. The aggressiveness with which surgeon searches for membranes during membrane peeling can be influenced by the knowledge that vitreoschisis can occur either preoperatively or during surgery. A higher index of suspicion can also be decisive in the choice to use or not to use surgical adjuncts that could function like particulate suspensions, such as triamcinolone, or colored stains, such as trypan blue and indocyanine green.

#### 2.2.1. Vitreoschisis and ILM in Myopic Eyes

**Figure 2:**
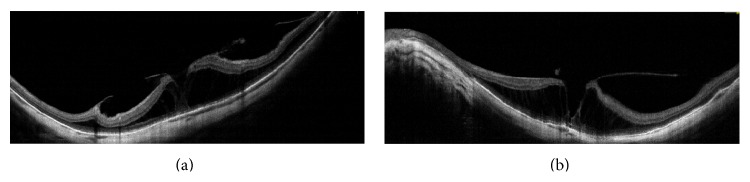
OCT images of myopic eye. Myopic foveoschisis associated with tangential traction due to ERM and vitreoschisis.

Myopia is associated with vitreous liquefaction excessive in relation to the degree of vitreoretinal adhesion, resulting in anomalous PVD and traction at vitreoretinal interface [13] (Figure 2). In high myopia, the anteroposterior axis is the longest, and the vitreous chamber may be prolate [32]. Meskauskas et al. suggested that elongation and enlargement of the vitreous chamber might increase the vitreous and retinal shear stress exerted by the movement of the eye, playing an important role in the pathogenesis of PVD and retinal detachment (RD) [33]. This shear stress could be the origin of the disintegration of the collagen network and the consequent vitreous liquefaction and PVD. The vitreous, partially liquefied, is more rapidly disrupted in larger eyes than in normal eyes [32]. This process leads to a significant reduction in the gel volume and a consequent increase in the liquid volume, which results in PVD or posterior vitreoschisis, whereas the increased rigidity of the preretinal vitreous might be the cause of undue tangential traction at the vitreoretinal interface either at the posterior pole or in the middle periphery [13, 32]. Furthermore, in highly myopic eyes, when PVD occurs, sheets of residual cortical vitreous often remain attached to the inner surface of the retina. These sheets may contribute to a number of vitreoretinal diseases due to their subsequent contraction [8].

In addition, in highly myopic eyes, an insufficient flexibility after axial length elongation could cause sclerotic retinal arteriole that determines a not distensible ILM [34]. The rigidity of the ILM seems to be the primary component that generates an inward tangential traction on the retina along the arterioles and may be responsible for the pathogenesis of myopic vitreoretinal diseases, such as foveoschisis, macular hole formation, and paravascular retinal break formation [34–36]. Sayanagi et al. [37] hypothesized that inflexibility of retinal vessels and the consequent tractional force in highly myopic eyes can lead to development of vascular microfolds. Bando et al. [38] identified fibroglial cell debris and collagen fibers on the inner surface of the ILM peeled from eyes with myopic foveoschisis in 70% of cases. The origin of these cells was not exactly determined; however, some cells such as astrocytes, abundant around retinal vessels, might migrate from the retina through small retinal pores in eyes with paravascular lamellar holes, produce collagen fibers, and initiate a proliferative response on the ILM. The rigid ILM, tightly attached to the posterior cortical vitreous, may cause the difficult differentiation between them on OCT images [39].

#### 2.2.2. Vitreoschisis in Diabetic Eyes

**Figure 3:**
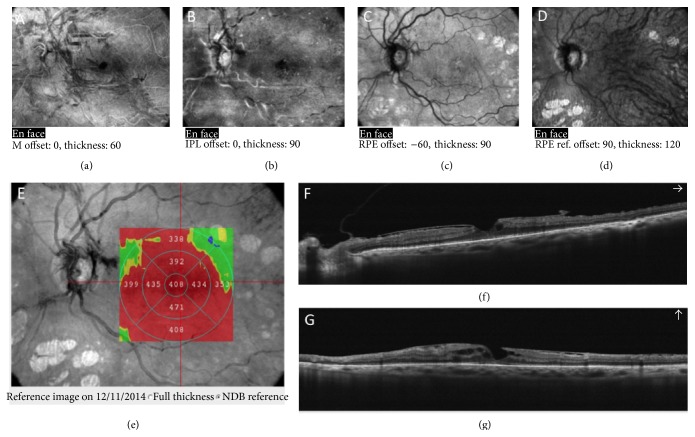
OCT images of diabetic eye. (a)–(d) En face OCT images at different segmentation thicknesses. (e) Increased retinal thickness map. (f)-(g) Vitreoschisis associated with vitreopapillary adhesion. The tangential traction induces wrinkling of ILM and intraretinal cysts.

The panmetabolic disease of diabetes mellitus (DM) induces structural and biochemical changes in vitreous tissue (Figure 3). The resulting diabetic vitreopathy plays an important role in the pathobiology of proliferative diabetic vitreoretinopathy [13]. Intravitreal glucose levels reflect blood glucose ones and can permit intravitreal nonenzymatic glycation reactions in patients with diabetes [40]: high levels of early glycation products were found in patients with diabetes when compared with patients without diabetes who undergo vitrectomy [41]. Functionally, advanced glycation end (AGE) cross-links in the collagen fibrils of vitreoretinal interface cause reduced solubility, tissue rigidity, a decreased susceptibility of proteins to enzymatic digestion, aggregation of collagen fibers, and dissociation of collagen from hyaluronan, resulting in vitreous destabilization and alterations of PVC and hyalocytes [40, 42]. Stitt et al. [40] suggested that AGE-derived cross-links on the vitreous collagen network may cause earlier age-related vitreous degeneration in patients with diabetes than in those without diabetes and may cause anomalous PVD, vitreoschisis, and vitreoretinal traction [40, 43]. Furthermore, proliferative diabetic retinopathy (PDR) alters the vitreous tissue by inclusion of fibrous tissue and vasogenic cells [43]. Structural changes at the vitreoretinal interface promote migration and proliferation of vasogenic cells in the vitreous and the consequent contraction can produce macular edema and vitreous hemorrhage [43]. Vitreoschisis in diabetics was first described by ultrasound [24] and by histopathology [25] in 80% of eyes with PDR. Spectral OCT-SLO studies [27] have furthermore detected vitreoschisis in half of eyes with macular hole and macular pucker. In a study of DME [44], 13 (57%) out of the 23 subjects with anomalous PVD had PDR. This is an important consideration, given the high prevalence of vitreoschisis in this group of patients [45]. In eyes with PDR, the cortical vitreous is rarely detached; rather there is a large, anterior, ring-like vitreoschisis, with multiple smaller ones, more posteriorly [46].

#### 2.2.3. Vitreoschisis and Surgical Outcomes

Induced PVD during pars plana vitrectomy (PPV) is a fundamental step to decrease the recurrence rate of RD. If the removal of PVC is incomplete, a thin but very adherent layer of the cortical vitreous remains attached to the retina and postoperative complications such as tractional redetachment and ERM reproliferation may occur. Indeed with an incomplete PVD mobile gel remains in the eye, with more room for it to exert dynamic traction, retinal shear stress, and redetachment; the free fluid enters the original or newly formed break at the point of residual vitreoretinal adhesion [46].

Vitreoschisis represents a convincing explanation for the recurrence of MH and MP after surgery. Indeed, if only the anterior layer or inner wall of the vitreoschisis cavity is removed during surgery, the posterior layer, or outer wall of the vitreoschisis cavity, will remain on the anterior surface of the retina.

In some studies of MH surgery, aggressive chromodissection [22] has been associated with a lower rate of recurrent disease [29, 47], which is likely because aggressive chromodissection of the vitreoretinal interface results in definitive removal of the outer wall of the vitreoschisis cavity. Other studies have reported a more favorable outcome by employing “ILM peeling” in MP surgery in comparison to those without aggressive membrane dissection [48].

Furthermore, Lois et al. in the FILM study have proved that ILM peeling, compared with no-ILM peeling, is more effective in reducing the risk of reoperation in patients with idiopathic stage 2 or 3 full-thickness macular holes (FTMHs), removing completely the potential tangential ILM traction and any residual cortex on ILM. However, they did not find any significant differences in distance visual acuity after the two techniques [49].

Triamcinolone acetonide (TA) is most commonly used as an adjunct to visualize vitreous, posterior hyaloid, preretinal membrane, and ILM during vitrectomy or chromovitrectomy. The granules of TA adhered to the residual posterior hyaloid, making it more visible [50]. This technique can disclose the residual hyaloid cortex pattern after surgical PVD and permit intraoperative visualization of vitreoschisis. Indeed, diffuse posterior hyaloid cortex is frequently found in high myopia and diabetic retinopathy, and an island-like cortex is often left on the macula, which can lead to future macular pucker. TA-assisted vitrectomy facilitated removal of residual island-like cortex, thus ensuring a low reproliferation rate on vitreomacular interface.

Recently, ocriplasmin has showed a proteolytic activity against fibronectin and laminin. The aim of the use of ocriplasmin is to cleave vitreoretinal interface with an intravitreal injection, causing a complete PVD [17]. Recent studies have shown that intravitreal injection of ocriplasmin can induce vitreous liquefaction and its separation from the retina [51–53], leading to closure of macular holes and resolution of VMT without causing serious adverse events [54, 55]. Stalmans et al. [56] conducted two pivotal phase III trials (TG-MV-006 and TG-MV-007) with a single injection dose of 125 *μ*g in patients with symptomatic VMA/VMT. Whereas ocriplasmin was generally well tolerated in these trials, recent studies showed some adverse effects, such as incomplete VMT release, retinal breaks, and visual impairment associated with subretinal fluid [57–59]. It is possible that ocriplasmin may have an enzymatic effect not limited to areas of VMA but diffuse on the retinal pigment epithelium or photoreceptors producing a phenomenon defined as lucency, characterized by the presence of subretinal fluid in macular area. Rod photoreceptors seem to be more susceptible to the effects of ocriplasmin than cone photoreceptors [60]. Some variables may limit the success of ocriplasmin: lens status, broad versus focal vitreomacular attachments, multiple vitreomacular attachments, and patient's age [58].

## 3. Vitreopapillary Adhesion

**Figure 4:**
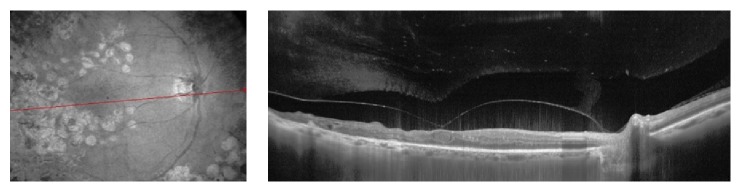
VPA associated with vitreomacular adhesion in diabetic retinopathy. Clear evidence of posterior precortical vitreous pocket at enhanced HD line scan.

Vitreopapillary adhesion is defined as a prominent vitreous membrane attached to the borders of the optic disc [61], associated with the development of PVD [62] (Figure 4). Dynamic VPA, which is associated with PVD stages 1 through 3, is occasionally severe enough to cause visual symptoms and anatomic changes [63, 64]. Affected patients are usually asymptomatic or report transient photopsias and gaze-evoked amaurosis. Visual acuity and automated perimetry are typically normal or almost normal. Rarely, a mild relative afferent pupillary defect may be transient [63, 64]. However, the resolution of symptoms occurs with progression to complete PVD [62]. Biomicroscopic signs often include fullness, elevation, and even subtle whitening of the peripapillary nerve fiber layer, which can simulate nontractional disc edema. Intrapapillary and peripapillary hemorrhages may occur [65], but they are benign and self-resolving [66]. The most common associated hemorrhages have been reported to be subretinal [67]. Although these hemorrhages can occur at any age, they are commonly associated with partial PVD development in young myopic patients [66].

The strong and persistent adhesion of the posterior hyaloid to the optic nerve head results in traction and tenting of the papillary rim as well as elevation of the optic nerve head, which appears as a pseudopapilledema upon examination of the fundus [1, 68]. The presence of an elevated optic nerve head must be differentiated from several etiologies of disc edema, such as papillitis, papilledema, optic nerve head drusen, optic nerve infiltration, and optic nerve or orbital masses [63, 68, 69].

This condition can cause possible optic nerve dysfunction [70]. Indeed, tractional forces elongate the retinal nerve fibers and inflect the central retinal vessels. The stretching, thinning, and consequent deformation of the ganglion cell axons reduce the anterogradely or retrogradely axoplasmatic flow and account for a sensory blockade of neuroretinal signals; the visual evoked potentials can result in alterations. Mechanical restriction and feeding of the central retinal blood vessels also decrease prelaminar blood flow [1]. In addition, optic atrophy might occur with established optic disc traction [1].

VPA is also known to induce epiretinal traction and intraretinal changes such as cysts, MHs, MP, macular edema [71], and age-related macular degeneration (AMD) [70] and to promote retinal and optic disc neovascularization [13].

While anomalous PVD may be the initial event, persistent adherence of the vitreous to the optic disc may influence the vectors of force that are exerted on the macular interface [72]. After anomalous PVD with vitreoschisis [8], the outer layer of the splitted PVC remains attached to the macula. In the absence of VPA, inward (centripetal) tangential traction likely throws the underlying retina into folds, resulting in MP. If the vitreous is still attached to the optic disc, the vectors of force could be changed, resulting in outward (centrifugal) tangential traction that induces central retinal dehiscence, MH [72], and/or ERM [73].

The vectors of force that result from VPA may conceivably contribute to the perifoveal vitreous detachment proposed by Johnson and associates [74] as the primary pathogenic event in the formation of macular holes.

**Figure 5:**
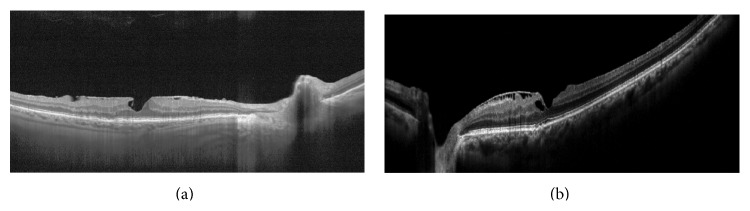
(a)-(b) VPA with pseudomacular hole (PMH).

Sebag and colleagues have demonstrated that VPA is significantly more common in FTMHs than in LMHs, MP, dry AMD, and age-matched controls. They also showed that VPA, when present in MHs and/or in MP, is highly associated with intraretinal cystoid spaces. Indeed, in their study, cysts were found in 100% of the eyes with VPA and MH, 80% of the eyes with MP and VPA, 75% of the eyes with LMH and VPA, only 42.9% of the eyes with LMH without VPA, and 4.3% of the eyes with MP without VPA [61]. Therefore, VPA is far more common in MP and MH with cysts as compared to LMHs or MP without cysts [61]. Foveal cysts are believed to be the precursors of either FTMHs or LHs [75, 76]. Wang and colleagues found that 100% of the eyes with MH and VPA were associated with intraretinal cysts. MHs and LMHs may have more cysts compared with MP because the inner retinal layers are severely disrupted by changing fluid dynamics [72]. VPA is also more frequent in LMHs than in the presence of pseudomacular holes (PMHs) [73] (Figure 5).

Van Newkirk and colleagues [77] detected vitreous attachment to the peripapillary retina in 65 of 65 patients with stage 3 MHs. The results of these studies suggest that LMHs represent an intermediate stage in the process of macular hole formation and that foveal pseudocysts with partial PVD become LHs if the base is conserved and become FTMHs if the outer retinal layer is disrupted [75]. These results have been confirmed by Wang et al. who discovered VPA in 87.5% of the eyes with FTMHs as compared with 36.4% of the eyes with LMHs and 17.9% of the eyes with MP [72].

Vitreopapillary traction syndrome has also been described, but it is usually in conjunction with other manifestations of anomalous PVD and includes rhegmatogenous RD retinal detachment, MP, MHs, and proliferative diabetic vitreoretinopathy [63]. Vitreopapillary traction can occur in the absence of other forms of anomalous posterior vitreous detachment in diabetic vitreoretinopathy [63].

The diagnosis of VPA is important because affected patients may inappropriately undergo ancillary testing, such as neuroimaging, or invasive procedures, such as lumbar puncture and referrals for retinal and neuroophthalmic evaluations. B-scan ultrasonography and OCT are valuable diagnostic tools that can be used to confirm the presence of vitreopapillary traction and to distinguish it from other causes of optic nerve elevation [65]. On B-scan ultrasonography, peripapillary vitreoretinal traction appears as a partial separation of the posterior hyaloid [68]. OCT can provide a precise diagnostic evaluation of the underlying etiology that may be challenging to establish with clinical observation and B-scan ultrasonography [78]. OCT image of peripapillary vitreoretinal traction is visualized as a partially detached vitreous band with continuous adherence to the optic nerve, thus causing elevation [69, 78].

### 3.1. Vitreopapillary Adhesion and Surgical Outcomes

The evaluation of OCT is important not only to determine the prognosis but also to identify the requirement for surgical intervention with PPV. Surgical release of VPA may significantly improve the anatomical and visual outcome [1, 73].

The tangential traction induced by VPA can cause damage to the outer retinal layers and the progression of a LMH. In presence of a LMH, VPA is considered as negative functional prognostic factor [73]. For these reasons, it is necessary to monitor LMHs, especially type 3, with OCT exam. Type 3 LMHs are the consequence of a vitreoschisis posteriorly to the level of hyalocytes and these holes can progress mainly in the presence of VPA.

In presence of preoperative VPA, the induction of PVD during PPV should be performed with caution because removal of the adherent peripapillary membranes or posterior vitreous may lead to iatrogenic excision of axons that compromise visual acuity and the visual field [79]. In addition, the suction for vitreous aspiration can induce iatrogenic retinal breaks [79].

## 4. Epiretinal Membrane

Epiretinal membrane is a common disease of the vitreoretinal interface. It is more commonly diagnosed in elderly people [80]; indeed, its prevalence is 2% in patients under the age of 60 years and 12% in those over 70 years of age [81]. It has been shown that the prevalence of ERM is lower in the Asians than in Caucasians [80–83]. A recent study demonstrated that ERM is significantly more common in the Chinese compared to Caucasians, Blacks, and Hispanics [84].

The ERM is a sheet of avascular fibrocellular tissue that can be formed on the ILM. This membranous tissue is usually deposited on the macula and then on the retinal periphery. ERM is classified as idiopathic (iERM) when it is not associated with any ocular disease and as secondary when it occurs during ocular processes such as RD, intraocular inflammation, trauma, retinal vascular diseases, and retinal surgery [85].

The term ERM refers to a group of vitreoretinal diseases that includes the following: MP [86], cellophane maculopathy, and epiretinal fibrosis [87]. However, many authors suggest that these diseases correspond to different stages of the same pathology [88]. The difference between the various stages has been noted in terms of the type of collagen and the thickness: collagen type VI characterizes cellophane membrane, which is the thinnest; types I and II characterize epiretinal fibrosis, while type IV and laminin are ubiquitous [88]. In a fundoscopic exam, ERM can appear as a translucent, semitranslucent, or opaque membrane (especially in later stages) that is located on the inner surface of the retina.

ERMs are composed of two main components: extracellular matrix (consisting of collagen, laminin, tenascin, fibronectin, vitronectin, and thrombospondin, among others) and retinal and extraretinal cells. A wide variety of cell types have been found in the membranes: glial cells (including microglia, Müller cells, and fibrous astrocytes), epithelial cells from the retinal pigment epithelium (RPE) and ciliary body, hyalocytes, blood-borne immune cells, fibrocytes, and myofibrocytes [85, 89].

An ERM can be static or it can develop over time. Histologically, two types of ERM can be observed: a simple one and a complex one [17]. The simple type, which grows directly on the ILM, is composed of a monolayer of retinal glial cells that produce type IV collagen (laminocytes) [90]. These cells for the first time described by Foos [91] like accessory glial cells migrating from the nerve fiber layer then were termed laminocytes by Snead et al. in spite of their laminar arrangement, close association with the ILM, and evidence of novel ILM production [90]. Laminocytes of the simple type show positivity to glial fibrillary acidic protein (GFAP), atypical protein of the astroglia, and the cytokeratin marker AE1/AE3 using immunocytochemistry [90]. The expression of GFAP indicates the ability of these cells to proliferate and migrate on the retinal surface. The complex or tractional type contains cells such as fibrous astrocytes, myofibroblasts, fibrocytes, hyalocytes, macrophages, and RPE cells in addition to glial cells [17, 90–93]. This multilayer of cells is separated from the ILM by a layer of native vitreous (type II) collagen that remains after incomplete PVD [92, 93]. The presence of type II collagen positivity in these cells suggests an additional role in secreting collagen into the vitreous gel. Simple ERM is a noncontractile type and is associated with mild to no visual symptoms. However, the contraction of myofibroblasts within the more complex type has been proposed to exert a progressive tangential traction at the vitreoretinal interface, which can result in retinal puckering, radial wrinkles, thickening, folding, or detachment, together with vascular distortion and retinal edema or a pseudohole. Thus, contractile ERM can reduce visual acuity and cause metamorphopsia [93–96].

Zhao et al. [97] stated that Müller cells and hyalocytes constitute the predominant cell type of the macular pucker. Myofibroblasts, the major cell type in complex ERM with glial cells [85], are considered to be derived from the transdifferentiation hyalocytes, REP cells, and glial cells. Indeed, many new studies have demonstrated high levels of nerve growth factor (NGF) and transforming growth factor *β*1 (TGF *β*1) in the vitreous. These growth factors can cause fibroblast migration and deposition, differentiation into myofibroblasts, and contraction of the extracellular matrix [85, 98]. The transdifferentiated cells are characterized by a downregulation of GFAP and cytokeratins, while proteins involved in motility and proliferation such as *α*-smooth muscle actin are upregulated [89]. Therefore, the GFAP content in epiretinal tissues has been shown to correlate inversely with the clinical contractility and directly with tractional forces [89].

**Figure 6:**
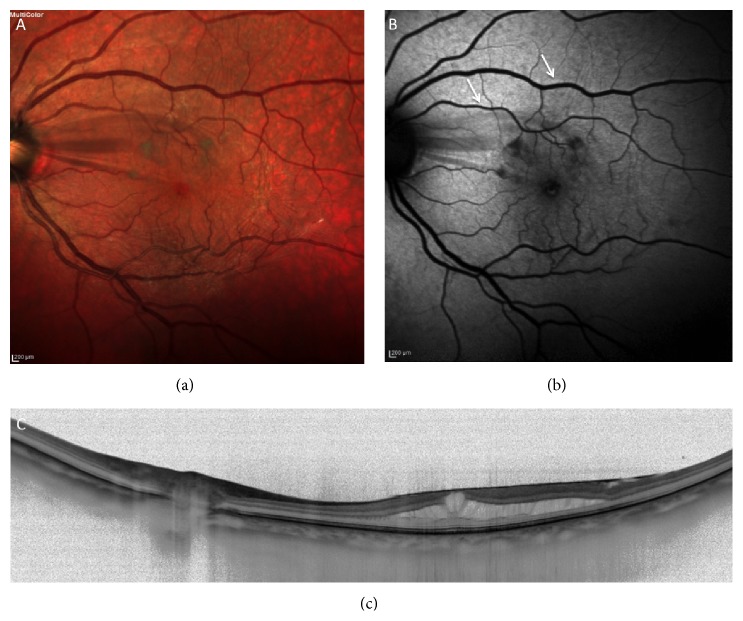
(a) The multicolor fundus photograph shows an iERM (a) with presence of “retinal vessel printings” (b) at fundus autofluorescence induced by tractional forces. The hyperautofluorescent lines (arrows) indicate the original location of the retinal vessels. (c) OCT scan of the same patient shows the cystoid macular edema induced by tractional forces.

The retinal changes induced by tangential traction may appear on the fundus examination as follows: irregular wrinkling, nerve fiber layer dragging, ectopic fovea, winding corkscrew vessels surrounding the overlying ERM, or major vessel straightening and crowding [99]. Fundus photography and, in particular, red-free or blue-reflectance imaging, can highlight the presence of ERM [17]. Fundus autofluorescence shows hyperautofluorescent lines that indicate the original location of the retinal vessels, which have been displaced because of a tractional ERM. These lines are called “retinal vessel printings” (RVP) and their visualization in eyes with ERM may give useful information about the severity and direction of tangential traction (Figure 6). Recently, Dell'Omo and colleagues proved that the presence of RVP is associated with a higher degree of irregularity of the external limiting membrane (ELM) and the junction between the photoreceptor inner segment and outer segment (IS/OS line) at the fovea and a higher average metamorphopsia score [100].

### 4.1. Posterior Vitreous Detachment and Epiretinal Membrane

The pathogenesis of iERM remains unknown despite significant progress in the field. The past theories have proposed that PVD certainly plays a critical role in the pathogenesis of this pathology through different possible mechanisms [101]. Among these, transient vitreoretinal traction during the development of PVD may cause breaks in the ILM through which glial cells and RPE cells can migrate and proliferate on the inner retinal surface [85, 102]. In addition, iERM may also result from the proliferation and transdifferentiation of hyalocytes contained within vitreous cortical remnants on the retinal surface following PVD [31, 103] and macrophages from subsequent inflammatory processes [102]. Indeed, partial or complete PVD has been found in 80% to 95% of eyes with idiopathic ERM [96, 101]. Actually, Foos and Bellhorn reported the presence of vitreous collagen fibrils in premacular fibrosis and within the ERM in their studies [91, 104]. Meanwhile, Kishi and Shimizu [101] reported the presence of defects in detached posterior hyaloid membranes of patients with idiopathic preretinal fibrosis. Sebag later clarified the pathogenesis with his concept of “anomalous PVD” [8]. This condition leads to a vitreoschisis and then to an ERM, especially when the split occurs anteriorly to the level of hyalocytes [8, 17, 26]. In this way, hyalocytes attached to the retina stimulate glial cells to proliferate upon an intact ILM to form the scaffolding that allows the uptake of other cells into the membrane [85, 93]. PVD and chronic irritation of glial cells can induce a local release of factors that induce cell gliosis, cellular hypertrophy, and upregulation of GFAP (simple static ERM) [105]. Andjelić et al. found Nestin-1 positivity, marker of progenitor cells of the retina, and Sox2 positivity, marker of epithelial stem cells and of pluripotency potential, to indicate the origin of transdifferentiated cells of ERM. When glial and pigment epithelial cells transdifferentiate to other cells, there is a reduction in cell-specific proteins such as GFAP and cytokeratins [105].

In addition to increasing age and PVD, other risk factors for this pathology are the presence of diabetes and hypercholesterolemia [84].

ERM is only symptomatic if the macular or perimacular area is involved. The initial formation of this epiretinal tissue does not usually cause any clinically important reduction in vision; however, the progression and contraction could be slow. Therefore, advancement of the disease results in significantly reduced visual acuity [106]. Although the cellular mechanism underlying visual impairment in this pathology remains unclear, an increasing number of studies have suggested that it is related to abnormalities in macular morphology caused by ERM traction. The contraction affects the outer segments with distortion or detachment of the retina and/or photoreceptors, and it disturbs the spatial arrangement of the cones [107]. Arichika et al. examined retinal changes in iERM and discovered that thickening was greater in the foveal region than in the extrafoveal regions and that the thickening of the external foveal retina to the inner plexiform layer was associated with visual impairment [106].

ERM can occur together with other diseases associated with the vitreoretinal interface.

Tangential centripetal traction on the fovea results in a cleft between the inner retina and outer retina; alternately, it may induce avulsion of the foveal tissue or the roof of a pseudocyst, leading to the formation of lamellar macular defects [108].

The spontaneous separation of ERM is uncommon. Nomoto observed 5 cases of spontaneous separation of iERM in 92 eyes [107].

**Figure 7:**
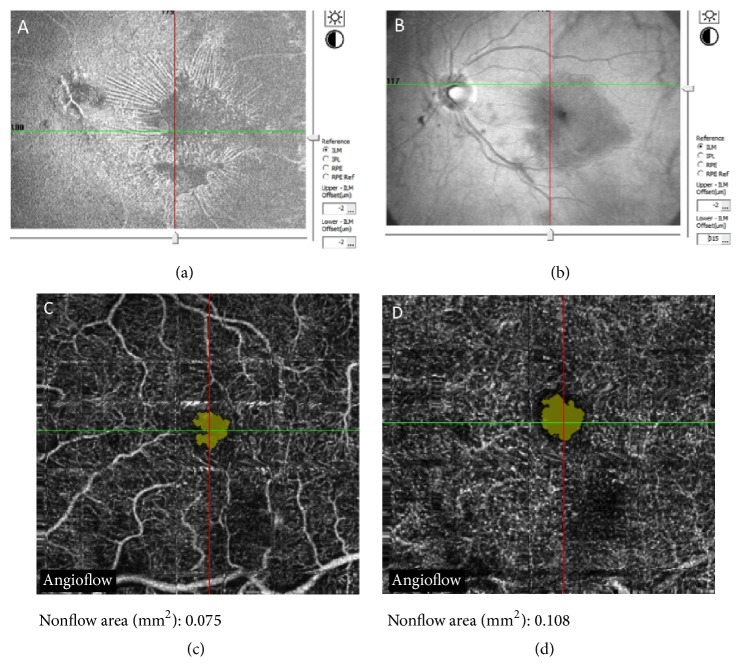
(a)-(b) iERM on en face OCT images at different segmentation thicknesses; at 315 microns offset from ILM the traction disappears (b); (c)-(d) angio-OCT images of retinal superficial plexus (c) and retinal deep plexus (d).

OCT is very useful in identifying the precise shape and size of ERM [14], in confirming the relationship between PVD and ERM, and in following its natural history [96] (Figure 7). Using OCT, it is quite easy to differentiate the posterior hyaloid, a minimally reflective signal, from an ERM, which is highly reflective [109]. However, so far, we do not have any classification of tangential traction, but only the classification of the morphology changes induced by anteroposterior vectors of traction [110].

OCT could also be shown to discriminate secondary from idiopathic types of abnormalities; in contrast, secondary ERM demonstrates focal adhesion points [111]. The retinal structural changes can be resolved after surgical removal of ERM, and some OCT parameters, such as macular thickness, are associated with the surgical outcome [97]. In recent studies, prolonged macular traction has been shown to cause irreversible photoreceptor cell loss and alignment disruption [112]. Outer retinal structures rarely return to normal after they are impaired, thus indicating a poor visual prognosis [113]. Therefore, prompt surgical intervention would be beneficial to prevent such damage [112].

### 4.2. Epiretinal Membrane and Surgical Outcomes

Falkner-Radler and colleagues, using spectral-domain OCT, identified the following prognostic factors in the ERM surgery: baseline visual acuity, the IS/OS line integrity of the junction between the photoreceptor inner segment and outer segment, the ILM profile, and foveal contour-like [114].

PPV with membrane peeling using vital dyes is the standard surgical procedure for patients with symptomatic ERM [114]. However, iERMs recur in approximately 10% of cases, and reoperation is required in approximately 3% of cases [115]. Sandali and colleagues showed a recurrence of 5% in 440 patients. They also demonstrated that ILM peeling seemed to be the only factor preventing ERM recurrence and that the use of staining dyes did not reduce the recurrence rate compared to ILM peeling in the absence of dyes [116].

Kenawy and colleagues have demonstrated that ERMs alter the cleavage plane during ILM peeling. Their study has shown that the formation of ERMs involves epiretinal glial proliferation with neuronal or glial cells on the retinal surface of the ILM and consequent intraretinal displacement. The adhesion of cells to the retinal surface of ILM might lead to focal force transmission into the retina during peeling with consequent retinal breakages and damage of Müller cells [117]. Such iatrogenic retinal damage after ERM-ILM peeling leads in patients with DME prominent cyst characteristics to the “floor effect.” Such phenomena consist in the collapse of retinal layers, damage of outer retina, and worsening of visual acuity [118].

Looking through the literature we found a further classification of tangential tractions, on which we are working on, based on the area and the depth of traction, was necessary. These features are better investigated with en face OCT analysis. The right interpretation of the forces involved in the epiretinal tangential tractions helps in a better definition of diagnosis, progression, prognosis, and surgical outcomes of many vitreoretinal diseases.
